# Editorial

**Published:** 1855-01

**Authors:** 


### Editorial.

---

### DR. BEALE'S CASE.

We have given much room to the able and interesting article by E. Hartshorne, M. D., on the Psychological effects of Ether. The article was written with reference to the medical and legal bearing of the case of Dr. Beale, and presents the case so well that, although long, we prefer to give it entire. Our space, however, forbids the publication of the article by Dr. Morton Stille, which is, indeed, an appendix to the other, and we may give it in our next No.

We are sorry that such a case has ever occurred ; but having occurred, whether Dr. B. is innocent or guilty we feel that, notwithstanding there is so much about the case which is repugnant to the better feelings of our readers, and such as we should be ever glad to exclude from our pages, yet the interest of the Profession and the public good demands a fair and thorough examination of the case.

Dr. B., if guilty, suffers very justly, and, indeed, the penalty does not begin to meet the magnitude of the crime. If innocent, no compensation can ever suffice for the injury. These circumstances would appear to demand more than ordinary care and patience in the legal tribunal, whose judgment must, to one or other of the parties, bring so much misery.

We ask now, What is necessary, in point of fact, to convict Dr. B. of the crime laid to his charge ?

It is answered : Miss M—— being of unimpeachable character, and her testimony being positive. This is all sufficient, and especially as no sinister motive can be conceived which would influence the testimony in such a case. In granting this for the prosecution, we should ask what is equally fair in return, viz : that the circumstances should render the case possible, and if any doubts should hang around the case, these should have the advantage of at least an examination.

We then first take the evidence of Miss M. for this in full—we refer to the article we publish from the pen of Dr. Hartshorne. Here we see the testiomny very positive—let us also examine if circumstances render it possible. To determine this we must take into consideration the length of time a person will remain under the influence of Ether when it has not been given to a state of unconsciousness or insensibility, we will say three minutes ; this we may however say, is an unusual length of time, but seldom more than one minute will expire before abilty to walk and talk are manifested. But what must have taken place during this eventful period ? First, the Dr. feels the pulse several times to see if all is right. Then he passes his hand up the arm ; next he encroaches on the fair and tranquil domain of her bosom—how long he lngers here is not known. We do contend, however, that, during the delay, more Ether must have been given, or some equally potent agent aroused to keep up that calm and fatal slumber. He, however, leaves this position ; passes round in front and raises her clothes ; feels her person ; and by this time, it appears from the testimony, her "memory" became "perfectly distinct." The effect of the ether is passing off, for sensation and consciousness are becoming "perfectly distinct." She even feels the breath of the Dr., but so etherial are her sensations that she does not feel or realize what part of his person is touching her, and, but for that dreadful pain, all might have been an illusion. Now we deny that all this could have taken place without the repeated inhalation of ether ; or that she was able to resist and hallow even as loud as when her tooth was afterwards extracted. Any other explanation would crowd more into so short a period of time than is possible, or make of the whole affair a very *sheepish* concern. She was perfectly conscious of every thing passing on around her ; she felt his breath, and yet says nothing about any more ether being administered. But—*yes but*—the whole affair appears to be real. It is true to nature ; yes, as true as if no ether was about it. How, then, shall we account for it ? Can it be an illusion ? It is proven by undisputed medical testimony that illusive dreams do take place very often when under the effects of ether ; and we believe that what the mind particularly grasps when passing under the influence of ether, that this it will retain until its effects pass somewhat off. The Profession so well understands this, that usually an effort is made to get the mind off of the operation about to be performed. Was Miss M. particularly liable to such an illusion at that time? Circumstances prove this beyond a doubt. First, she is accompanied to the office by her betrothed ; and, second, it is the very day on which the menstrual discharge is to take place. A determination of blood is already directed to the parts, and may not, will not, the ether increase this? The circumstances then are favorable, and we are obliged to regard them as such. If virgin innocence could have such a dream, this is certainly a favorable case. What was done to disprove this illusion? Positively nothing. The mere fact of blood being about her person immediately after she left the office, is no evidence at all—it may have been the regular monthly discharge. If the liberties about her bosom and person might be an illusion, equally certain might the *pain* be of the same character. It is merely the last link in the chain, and that this *link* had been broken should most certainly have been proven. Dr. B. had surely a right to demand this. It could not have made his case worse ; it might have knocked his *chains* from his prison door, and bid him go forth a free and unsullied man. It might also have restored to maiden virtue a charm which no etherial illusion could have taken away.

But we are saying much more on this subject than we intended. The article of Dr. Hartshorne is so full and complete, that but little more need be said. From the evidence before us we must belive that Msss M. was either in not a fit state to give reliable testimony, and hence every statement should be, by the best of medical evidence, sustained, or she proves herself in a situation capable of resistance. Her unimpeachable character, and the notoriety which such a charge against Dr. B. would expose her, must forbid the latter supposition.

We hope the Profession will have learned a lesson or two from this case.

One is to feel the responsibility which rests on them in the administration of such an agent for the extraction of teeth ; and second, refuse to administer it, unless properly supported by the patient's friends, and remunerated for the responsibility and trouble.

We cannot but feel such solicitude for our patients, while under the influence of ether or chloroform, that such a case as that of Dr. B. cannot be regarded as real. We very frequently administer the chloric ether ; but very seldom without a friend is present. We have had many cases of illusive dreams—sometimes that we were pulling their teeth, and in such cases the scream and struggle accompanies the operation. We have then repeated the dose, drawn the mind from the operation, and perfect composure was the result. In the first instance pain was contended for as real ; in the second, no pain was felt.

Hundreds of times have we administered these agents to relieve pain, but in no instance have we witnessed improper conduct by the ladies, or heard improper language.

---

*A Dictionary of Medical Terminology, Dental Surgery, and the Collateral Sciences.* By C. A. Harris, Professor of the Principles of Dental Surgery, in the Baltimore College, etc.

We have before us the second edition of this work, just from the press. By glancing over it we find the author has made a very material change in the plan of the work ; casting out the biographical department entirely, and substituting instead about eight thousand more words, with complete definitions.

All new terms, and recent discoveries in dental science, are introduced into the present work. In it we find, too, all the ordinary medical terms, with full definitions.

With this work at hand, the dental student would but seldom, if ever, have occasion to refer to the ordinary medical dictionary : and it is much more valuable to him since it contains all the special terms of his department. It is valuable in another respect. It presents, in a condensed and concise form, the leading principles of all the operations in each department of dental science ; and these, too, up to the present day. This work *is* up with the times.

This work is not only valuable to the dental student and practitioner, but for the physician it has a store that he will not find elsewhere. It should have a place in the library of *every* physician. Here is for him a clear and concise view of the principles of one important department of medical science ; and one, too, that has been almost wholly overlooked, or neglected by the great majority of physicians.

The work contains 800 pages, 8 mo. In regard to the style of the work, it is sufficient to say, that Lindsey & Blakiston of Philadelphia, are the publishers. It is for sale by Dr. J. M. Brown, corner of Fourth and Walnut sts., Cincinnati, O.

---

*Facts for the People, relating to the Teeth, showing their influence upon the Health, Speech and Looks, with directions for their care and preservation..* By T. D. Thompson, D. D. S.

This is the title of a very neat little volume, containing some 250 pages.

In glancing hastily over its pages it appears to be what its name imports; for we see many valuable facts which the people should know, and they are given in such a style and manner as will generally please. Indeed the whole book appears to be a grouping together of valuable facts; and although the professional dentist may not see much which is new and of special interest to himself, yet his patients, we doubt not, would think otherwise.

The work contains some twenty chapters, beginning with the early history of the science, and closing with the use of chloroform and ether; treating of the formation of the teeth; first and second dentition; cause of decay; neuralgia; tooth-ache; artificial teeth, etc., etc. The work is creditable to Dr. Thompson, and we hope the profession and the people for whom it is published, WILL CAREFULLY READ AND PROFIT BY THESE FACTS.

---

### NEW YORK TEETH MANUFACTURING COMPANY.

The gentlemen engaged in this enterprise appear determined to push their establishment to success, and we do not see why they may not. There appears to be no limit to the demand for not only teeth, but almost every other article used in dental practice. By reference to their advertisement, it will be seen that they keep a general assortment of materials. We believe the Dental Expositor will be published quarterly by this company, and hope to see it a valuable addition to our periodical literature.

---

### LOOMIS' BLOCK TEETH.

We publish, in the present No. of the REGISTER, a communication from Dr. Loomis on the subject of his improvement. We had supposed, that the Dr. had overcome, in a great measure, the shrinkage of the material. This was, we perceive, an error. He appears first to ascertain the amount of shrinkage in a given kind of body, and then make his calculations to meet this. We can readily see how this may be done for ordinary practical work; but that this should be so accurate as to give good suction sets of teeth, is a little unaccountable to us as yet.

Such an object is really worth laboring for; and yet the question comes up, Will not such operations, to be sufficiently strong, be clumsy in the mouth?

We have a specimen sent us by Dr. Loomis of a complete upper denture, which looks remarkably well, and in weight is not equal to many made on gold. We should fear, however, that a fall on the floor would be more apt to injure it than an operation put up on gold.

Dr. Loomis is certainly very sanguine, for he speaks confidently of success, and we are sure that we shall like to hear others speak just as confidently of the availability of his plan.

---

### MISSISSIPPI VALLEY ASSOCIATION OF DENTAL SURGEONS.

The members of this Society and the Profession, will please bear in mind that Wednesday 21st of February, 1855, at 10 o'clock A. M., is the time for the next meeting; this takes place in the lecture room of the Ohio College of Dental Surgery. We are thus explicit as it regards the time, for there is an error in the date as published in the last proceedings; Wednesday being on the 21st instead of 23rd, as then published.

We hope to see the Profession well represented. Those who are not members of the Association are always made welcome, and can be present at any of the meetings. If members would come in on Monday, they could also attend the meetings of the Ohio Dental College Association, which convenes on that day. Two or three essays have been sent in, in anticipation of the $100 prize, and we have no doubt the meeting will be an interesting one. We would call the attention of the members of the Association and the Profession, to the communication of Dr. A. M. Leslie, Chairman of the Committee on Dental Progress, and hope every aid will be given that the Committee may bring in an interesting report.

---

### THE OHIO DENTAL COLLEGE ASSOCIATION.

The Stockholders and other members of this Association are notified that Monday 19th of Febrnary, 1855, at 2 o'clock P. M., is the time of meeting. Since our last meeting a new college has been erected, and the Building Committee are anxious to have all the stockholders present, that they may give a full report of what has been done. They feel assured that the Association will feel it a privilege to convene, in a building which they may call their own. There will necessarily be much business to transact of an important character, and hence every share of stock should be represented, so that the interest of the Institution they oversee may be, in the best possible manner, subserved. It will be recollected that the commencement exercises of the College comes off on the evening of Wednesday 21st of February, at which time two or three addresses will be delivered and degrees conferred. We hope the Profession generally will feel at perfect liberty to attend.

---

### EGYPTIAN SPECIMENS OF DENTAL SCIENCE.

We see it recently stated, in one of our daily papers (*Enquirer*), that Mr. Finney, a dentist, late of Alexandria. Egypt, is said to have found a "stuffed tooth in a mummy, and several teeth in other mummies which bore marks of filling."

We are sorry, if this is true, that Mr. Finney has not made the facts public himself—not even the kind of filling is named—and we cannot but regard the report as unfounded. It has often been a matter of surprise that the tombs of Egypt have not furnished us with some evidence of the state of dental science, which should somewhat compare with the known progress made in medical science. Evidence of dental science will now outlast every vestige of frail mortality, even if preserved with Egyptian skill.

---

### GOLD FOIL.

We have recently been using some of Mr. R. H. Ransley's gold foil, which we are glad to commend as a good article. Mr. Ransley is successor to the late John King & Co., Philadelphia, and will, with promptness, fill all orders entrusted to him.

---

#### SUBSCRIBERS.

We sometimes wonder if those who read the REGISTER ever notice a little article headed as if specially directed to their attention. While there are some who punctually respond to these obtruding missives, yet we fear that a large majority never notice them. To such we wish to say that we have labored long and ardently for their benefit. "*Benefit*," says one, "you do not benefit me." But yet we persist benefit, and unblushingly say that there is not a member of the Profession but is more or less benefitted by our periodical publications; and it is all nonsense to meet the demands of dental science and yet not read its literature. We confess, reader, that if we were not benefitted professtionally, and did not feel that we were also benefitting the Profession, not even this No. would we send you. The fact is, there are none of us so perfect in our dental knowledge, and so thoroughly understand its practical detail, but that we may be benefitted by the suggestions of others, even if they are not so well posted as ourselves. But we wish to say to those who have not heretofore noticed the articles headed "*Subscribers*," that we hope they will look over the quarterly receipts, which we publish as accurately as possible, and see if pecuniarly we are profitted by our labor. It might be of some interest to some to look back for several years and see if they can find their own name in the list.

We are so well satisfied that many have no idea of their indebtedness, that we are preparing their accounts, and shall send them by mail as soon as we can. We hope, in all such cases, our letters will be promptly attended to. If any mistakes occur we shall, with pleasure, rectify them. But it cannot be expected we shall stop the REGISTER and remit our accounts where the REGISTER has been taken out of the office for a year. It would not do to set such precedents, and we cannot afford it. But we need not anticipate, for we have never had but two or three such cases, and believe there cannot be many more *such* in the profession.

---

#### CONTRIBUTIONS TO THE MUSEUM OF THE OHIO COLLEGE OF DENTAL SURGERY.

We are in receipt of some morbid specimens of teeth, sent by Dr. Merchant Kelly, of Indiana, for which we return our thanks. Dr. Duncan of this city, has also our thanks for a specimen of osseous union, caused by exostosis.

Messrs. Orum and Armstrong have sent us a beautiful card for our museum. It is a full set of gum teeth, put up in articulating model. They show, to a good advantage, the relative size of the teeth, the adeleptation of cusp to cusp, had the neat fit of gum, etc., etc. We think these gentlemen have improved on the strength of their teeth—their last assortment stand the heat very well.

### RECEIPTS FOR THE REGISTER.

| | | | |
|---|---|---|---|
| Dr. John Allen, vols. 5, 6 and 7 | 5 00 | Dr. John Fry, vol. 8 | 2 00 |
| Dr. Merchant Kelley, vol. 8 | 2 00 | Dr. S. P. Harington, vol. 8 | 1 00 |
| Dr. T. R. Willard, vol. 7 | 2 00 | Dr. R. C. Cypher, vol. 8 | 2 00 |
| Dr. T. H. Breswter, vols. 6 and 7 | 4 00 | Dr. E. S. Holmes, vol. 8 | 2 00 |
| Dr. Joseph Richardson, vol. 7 | 2 00 | Dr. M. Reynold, vol. 8 | 2 00 |
| Dr. Hiram A. Hartzell, vol. 8 | 2 00 | Dr. E. W. Kelser, vol. 8 | 2 00 |
| Dr. H. B. Young, vol. 8 | 2 00 | Drs. Hawkins and Ely, vol. 8 | 2 00 |
| Dr. Wm. Smith, vols. 5, 6 and 7 | 6 00 | Dr. S. Hinson, vol. 7 | 2 00 |
| Dr. Jesse Kelsey, vol. 8 | 2 00 | Dr. Eli Collins, vols. 7 and 8 | 3 62 |
| Dr E. McCuen, vol. 7 | 2 00 | Dr. B. T. Whitney, vol. 8 | 2 00 |
| Dr. H. E. Eames, vols. 6 and 7 | 4 00 | Dr. H. Peebles vols. 7 and 8 | 4 00 |
| Dr. W. D. Jenks, vol. 8 | 2 00 | C. E. Kells, vol 8 | 2 00 |
| Dr. E. J. Toof, vol. 8 | 2 00 | R. E. Cole, vols. 3 and 8 | 4 00 |
| Dr. J. H. Meredith, vol. 8 | 2 00 | A. J. Reeve, vol. 8 | 1 50 |
| Dr. E. S. Perkins, vol. 8 | 2 00 | | |

